# NF-kB overexpression and decreased immunoexpression of AR in the muscular layer is related to structural damages and apoptosis in cimetidine-treated rat vas deferens

**DOI:** 10.1186/1477-7827-11-29

**Published:** 2013-04-09

**Authors:** Juliana Y Koshimizu, Flávia L Beltrame, José P de Pizzol, Paulo S Cerri, Breno H Caneguim, Estela Sasso-Cerri

**Affiliations:** 1 Department of Morphology, Laboratory of Histology and Embryology, Araraquara Dental School-UNESP Univ. Estadual Paulista, Brazil; 2 Department of Morphology and Genetics, Federal University of São Paulo (UNIFESP) São Paulo/SP, Brazil

**Keywords:** Vas deferens, H_2_ receptors, NF-kappaB, Androgen receptors, Apoptosis, Morphometry

## Abstract

**Background:**

Cimetidine, histamine H_2_ receptors antagonist, has caused adverse effects on the male hormones and reproductive tract due to its antiandrogenic effect. In the testes, peritubular myoid cells and muscle vascular cells death has been associated to seminiferous tubules and testicular microvascularization damages, respectively. Either androgen or histamine H_2_ receptors have been detected in the mucosa and smooth muscular layer of vas deferens. Thus, the effect of cimetidine on this androgen and histamine-dependent muscular duct was morphologically evaluated.

**Methods:**

The animals from cimetidine group (CMTG; n=5) received intraperitoneal injections of 100 mg/kg b.w. of cimetidine for 50 days; the control group (CG) received saline solution. The distal portions of vas deferens were fixed in formaldehyde and embedded in paraffin. Masson´s trichrome-stained sections were subjected to morphological and the following morphometrical analyzes: epithelial perimeter and area of the smooth muscular layer. TUNEL (Terminal deoxynucleotidyl-transferase mediated dUTP Nick End Labeling) method, NF-kB (nuclear factor kappa B) and AR (androgen receptors) immunohistochemical detection were also carried out. The birefringent collagen of the muscular layer was quantified in picrosirius red-stained sections under polarized light. The muscular layer was also evaluated under Transmission Electron Microscopy (TEM).

**Results:**

In CMTG, the mucosa of vas deferens was intensely folded; the epithelial cells showed numerous pyknotic nuclei and the epithelial perimeter and the area of the muscular layer decreased significantly. Numerous TUNEL-labeled nuclei were found either in the epithelial cells, mainly basal cells, or in the smooth muscle cells which also showed typical features of apoptosis under TEM. While an enhanced NF-kB immunoexpression was found in the cytoplasm of muscle cells, a weak AR immunolabeling was detected in these cells. In CMTG, no significant difference was observed in the birefringent collagen content of the muscular layer in comparison to CG.

**Conclusions:**

Cimetidine induces significant damages in the epithelium; a possible antiandrogenic effect on the basal cells turnover should be considered. The cimetidine-induced muscle cells apoptosis confirms the susceptibility of these cells to this drug. The parallelism between enhanced cytoplasmic NF-kB immunolabeling in the damaged muscular tissue and muscle cell apoptosis suggests that this drug may avoid the translocation of NF-kB to the nucleus and interfere in the control of NF-kB-mediated smooth muscle cell apoptosis. The decreased immunoexpression of ARs verified in the damaged muscular tissue reinforces this possibility.

## Background

The presence of androgen receptors (ARs) in the epididymis and vas deferens has confirmed the importance of testosterone for the maintenance of an adequate microenvironment during sperm maturation [1]. In the vas deferens, these authors have demonstrated a weak and moderate expression of ARs in the principal and basal epithelial cells, respectively. In castrate mice, Hamilton *et al.*[2] demonstrated alterations in the epithelial cells of vas deferens such as absence of smooth endoplasmic reticulum, reduction in the size of the Golgi apparatus and decreased number of mitochondria. Therefore, these results indicate that androgens are essential for the structural and functional maintenance of the vas deferens epithelium.

ARs have also been detected in the smooth muscle layers of vas deferens [1]. The contraction of the muscle cells may be androgen dependent since androgenic hormones seems to play a role in the control of the calcium ions channels [3-5]. Additionally to testosterone, the muscular contraction of vas deferens is also dependent on histamine action. In rodents, the vas deferens muscular contraction has been inhibited by histamine [6,7]. This effect was confirmed after the evaluation of the effect of cimetidine (histamine H_2_ receptor antagonist) on the smooth muscle contraction of vas deferens. In this study, the inhibitory response of the muscular contraction was inhibited by this H_2_ receptor antagonist [8].

In the parietal gastric cells, cimetidine inhibits the acid secretion induced by histamine and has been used as antacid and anti-ulcer drug [9]. It has also been described that cimetidine presents an antiangiogenic effect in the ulcer granulation tissue. This effect has favored the treatment of cancer, suppressing the growth of several tumors by inhibiting tumor-associated angiogenesis [10,11]. The main adverse effects which have been reported in male patients treated at long term with cimetidine are: loss of libido, impotence [12], increased luteinizing hormone (LH), testosterone and prolactin levels [13,14] and ginaecomastia [15]. Most of these effects have been related to an anti-androgenic effect of cimetidine since antiandrogenic drugs leads to increased levels of LH due to desensitization of androgen receptors and, then, the inhibition of the negative feedback for gonadotrophin secretion [16]. The antiandrogenic effect of cimetidine was confirmed by other studies in which androgen receptors were competitively blocked by cimetidine in the pituitary and hypothalamus [17] and other tissues that require androgens [18,19]. In the rat testes, cimetidine impairs the seminiferous tubules, causing reduction in the diameter of the seminiferous tubules at androgen dependent stages, loss of germ cells by apoptosis [20,21], reduction in the number of Sertoli cells due to apoptosis [22,23], alterations in the peritubular tissue, including peritubular myoid cell death [21,22,24] and atrophy of the testicular microvasculature [25]. The impairment in the peritubular tissue has been associated to a direct toxic effect of cimetidine on the peritubular myoid cells [24]. Moreover, significant alterations in the testicular microvasculature have also been detected and related to cimetidine-induced vascular smooth muscle cells apoptosis [25]. In this study, this harmful effect of cimetidine on smooth muscle cells has been related to a possible antiandrogenic and/or H_2_ receptors antagonist effect since histamine and androgens play a function role in these cell types.

In addition to histamine and hormonal control, smooth muscle cells show a constant activity of nuclear factor kappa B (NF-kB), which promotes proliferation of these cell types [26]. NF-kB plays a key role in inflammation, immune response, tumorigenesis and protection against apoptosis [27-29] since it activates the transcription of many genes, some of which directly block the activation of caspases, involved in apoptosis [30]. Thus, NF-kB transcription and activation play a crucial role in regulating the process of cell death by apoptosis in several cell types including smooth muscle cells [26,31].

A recent study has reported that cimetidine inhibits the translocation of NF-kB into the nucleus, decreasing the transcription of antiapoptotic genes and inducing apoptosis in salivary gland tumor cell [32]. Thus, regarding the susceptibility of smooth muscle cells to the treatment with cimetidine, we purpose to evaluate the structural integrity of the epithelium and smooth muscular layer of the vas deferens in cimetidine-treated adult rats. Cell death by apoptosis, AR and NF-kB immunoexpression in the smooth muscle cells following cimetidine treatment were also evaluated.

## Methods

### Animals and treatment

Adult Holtzman male rats aging 100-day-old (300 g) were maintained at 25°C, standard lighting conditions (12-h light/dark cycle), fed laboratory rat chow and given water *ad libitum*. Principles of laboratory animal care and national laws on animal use were observed. The protocol of this study was authorized by Ethical Committee for Animal Research of the Dental School of São Paulo State University (UNESP-Araraquara). The animals from the cimetidine group (CMTG; n=5) received daily intraperitoneal injections of 100 mg/kg b.w. of cimetidine (Hycimet® 300 mg; Hypofarma, MG). In this study, cimetidine has been used to produce effects in the male reproductive tract in rodents; therefore, dosages were not selected to mimic human pharmaceutical use. The animals from control group (CG; n=5) received saline solution by the same route. The rats received the treatment for 50 days, period correspondent to the duration of spermatogenesis in adult rats [33].

### Histological procedures

The distal portions of vas deferens were fixed in 4% formaldehyde (freshly prepared from paraformaldehyde) buffered at pH 7.2 with 0.1 M sodium phosphate for 48 hours at room temperature. Subsequently, the specimens were dehydrated in graded ethanol and embedded in paraffin. The sections were stained with hematoxylin and eosin (H&E) and Masson´s trichrome for morphological and morphometrical analyzes. Sections stained by Picrosirius-red method [34] were analyzed under polarized light for evaluation of collagen content of connective tissue. The analyzes were performed under a light microscope BX-51 (Olympus).

### Morphometrical analyzes

In five non-serial cross-sections of vas deferens/animal, stained by Masson´s trichrome, the total area (μm^2^) of smooth muscular layer (at x70) and the perimeter (μm) of epithelial tissue (at x180) were measured by using an image analysis system (Image Pro-Express 6.0, Olympus).

### Picrosirius-polarization method and collagen content measurement

In the picrosirius-red stained sections, the collagen quantitative analysis in the muscular layer of vas deferens was performed in three non-serial sections per animal, totalling 15 sections per group. The sections were analyzed using an Olympus BX51 microscope equipped with filters to provide circularly polarized illumination. All image-acquisition parameters were standardized and the intensity of acquisition illumination was calibrated by adjusting only the microscope condenser aperture. The images were obtained with an x40 objective lens, recorded on a digital camera (DP-71, Olympus) and analyzed using ImageJ® image analysis software (http://rsbweb.nih.gov/ij/). The analysis methodology was performed according to Manni *et al.*[35]. Collagen content was calculated as a percentage of the area of each image (24,137μm^2^; 3,338,208 pixels). The images were loaded and colors were isolated by using the hue histogram filter available in "Threshold Colour". Images were subjected to a threshold so that each nonwhite pixel was turned black and each white pixel remained white. Then, the number of black pixels in each image was used to calculate the percentage of the image area that corresponded to a certain color. To determine the proportion of different colored collagen fibers, it was subtracted image into its hue, saturation and value components (also an automated function provided by the image-analysis software). Only the hue component was retained and a histogram of hue frequency was obtained from the resolved 8-bit hue images which contain 256 colors. The following hue definitions: red/orange 2–38 and 230–256; yellow 39–51; and green 52–128 [36] were used. The number of pixels within each hue range was determined and expressed as a percentage of the total number of collagen pixels which in turn was expressed as a percentage of the total number of pixels in the image.

### Statistical Analysis

A Jandel Statistical SigmaStat 2.0 software was used for the statistical analysis of the morphometrical data. The comparison between groups was performed by the one-way ANOVA followed by Student´s t-test; the significance level accepted was p≤0.05.

### TUNEL method

For detection of cell death, the TUNEL (**T**erminal deoxynucleotidyl-transferase mediated d**U**TP **N**ick **E**nd **L**abelling) method was performed as previously described [21] and according to the Apop-Tag Plus kit (Chemicon Internacional, USA). Sections adhered to silanized slides (3-aminopropyltrithoxysylane – Sigma-Aldrich Chemical Co., St. Louis, USA) were treated with 20 μg/ml proteinase K (Sigma- Aldrich Chemical Co., St. Louis, USA) and immersed in 3% hydrogen peroxide. After immersion in equilibration buffer for 20 min, the sections were incubated in TdT enzyme (Terminal deoxynucleotidyl Transferase) at 37°C for 1 hour in a humidified chamber. The reaction was stopped by immersion in a stop/wash buffer for 20 min and incubated in anti-digoxigenin-peroxidase in humidified chamber at 37°C for 30 min. The reaction was revealed with 0.06% 3.3'-diaminobenzidine tetrahydrochloride (DAB – Sigma-Aldrich Chemical Co., St. Louis, USA) and counterstained with Carazzi's hematoxylin. Sections of involuting mammary gland, provided by the manufacturer of the Kit, were used as positive controls for the TUNEL method. The sections used as negative controls were submitted to the same protocol, except the step of incubation in the TdT enzyme.

### NF-kB and AR immunohistochemistry

Paraffin sections adhered to silanized slides were immersed in 0.001 M sodium citrate buffer pH 6.0 and maintained at 90°C for 30 min in a microwave oven for the antigen recovery. The sections were treated with 3% hydrogen peroxide to inactivate endogenous peroxidase, washed in 50 mM phosphate-buffered saline plus 200 mM of sodium chloride (PBS) pH 7.3 and, then, were incubated with 2% BSA for 30 min. The sections were incubated overnight with the anti-NF-kB p65 (1:50; Abcam, Cambridge, UK; ab31481) or anti-AR (1:200; Chemicon-Millipore, USA; PG-21; 06–680) rabbit primary antibodies. Subsequently, the reaction was performed according to the LSAB-plus kit (Dako, USA); the sections were incubated for 30 min with biotinylated link universal antibodies at room temperature and, then, with streptavidin-HRP at room temperature for 30 min. After washes in PBS, the reaction was revealed by DAB (Biocare Medical; USA) and the sections were counterstained with Carazzi’s hematoxylin. For the negative controls, all the steps were performed following the same protocol, except that the step of incubation in the primary antibody was replaced by incubation in rabbit non-immune serum.

### Transmission Electron Microscopy (TEM)

Fragments of distal portions of vas deferens were fixed for 16h in a mixture of 4% formaldehyde (freshly prepared from paraformaldehyde) and 5% glutaraldehyde buffered at pH 7.2 with 0.1 M sodium cacodylate. After washings in 0.1M sodium cacodylate at pH 7.2, the specimens were transferred to cacodylate-buffered 1% osmium tetroxide at pH 7.2 for 1 h. Subsequently, the specimens were immersed in 2% aqueous uranyl acetate for 1 h, dehydrated in graded concentrations of ethanol, treated with propylene oxide and then embedded in Araldite. Semithin sections were stained with 1% toluidine blue and the suitable regions were carefully selected for trimming of the blocks. Ultrathin sections were collected on grids, stained in alcoholic 2% uranyl acetate and lead citrate and examined in a transmission electron microscope (Philips-CM 100).

## Results

### Light microscopy

**Figure 1:**
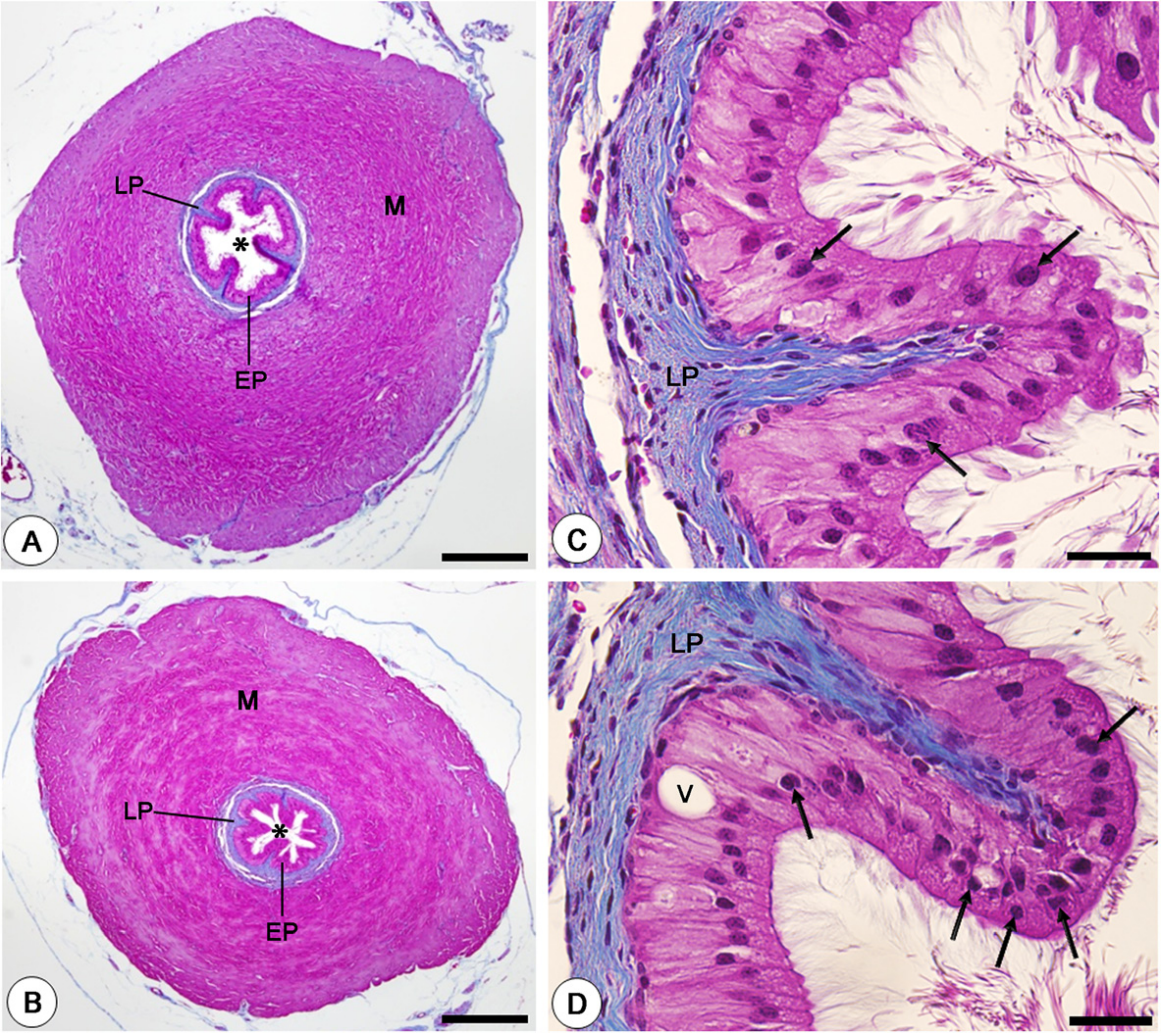
**Photomicrographs of vas deferens of animals from CG (A and C) and CMTG (B and D) stained by Masson's trichrome.** In **B** (CMTG), note that the epithelium (EP) is intensely folded and surrounds a decreased lumen (asterisk) in comparison to CG **(A)**; a reduction in the muscular layer (M) is also evident. Lamina propria (LP). In **C**, a high magnification of epithelium (EP) shows epithelial columnar cells with normal aspect (arrows). In **D**, pyknotic nuclei (arrows) and vacuolization (v) are observed in the epithelial cells. Bars: 340 μm **(A**, **B)**; 33μm **(C**, **D)**.

In the sections stained by Masson's trichrome (Figures 1A-1D), the vas deferens of animals from CMTG (Figure 1B) showed intensely folded epithelium surrounding a small lumen, and the muscular layer was reduced in comparison to CG (Figure 1A). The epithelium of CG showed apposed columnar cells with normal aspect (Figure 1C). Otherwise, in CMTG, the epithelial columnar cells showed pyknotic nuclei strongly stained by hematoxylin and intraepithelial vacuoles (Figure 1D).

### TUNEL labeling

**Figure 2:**
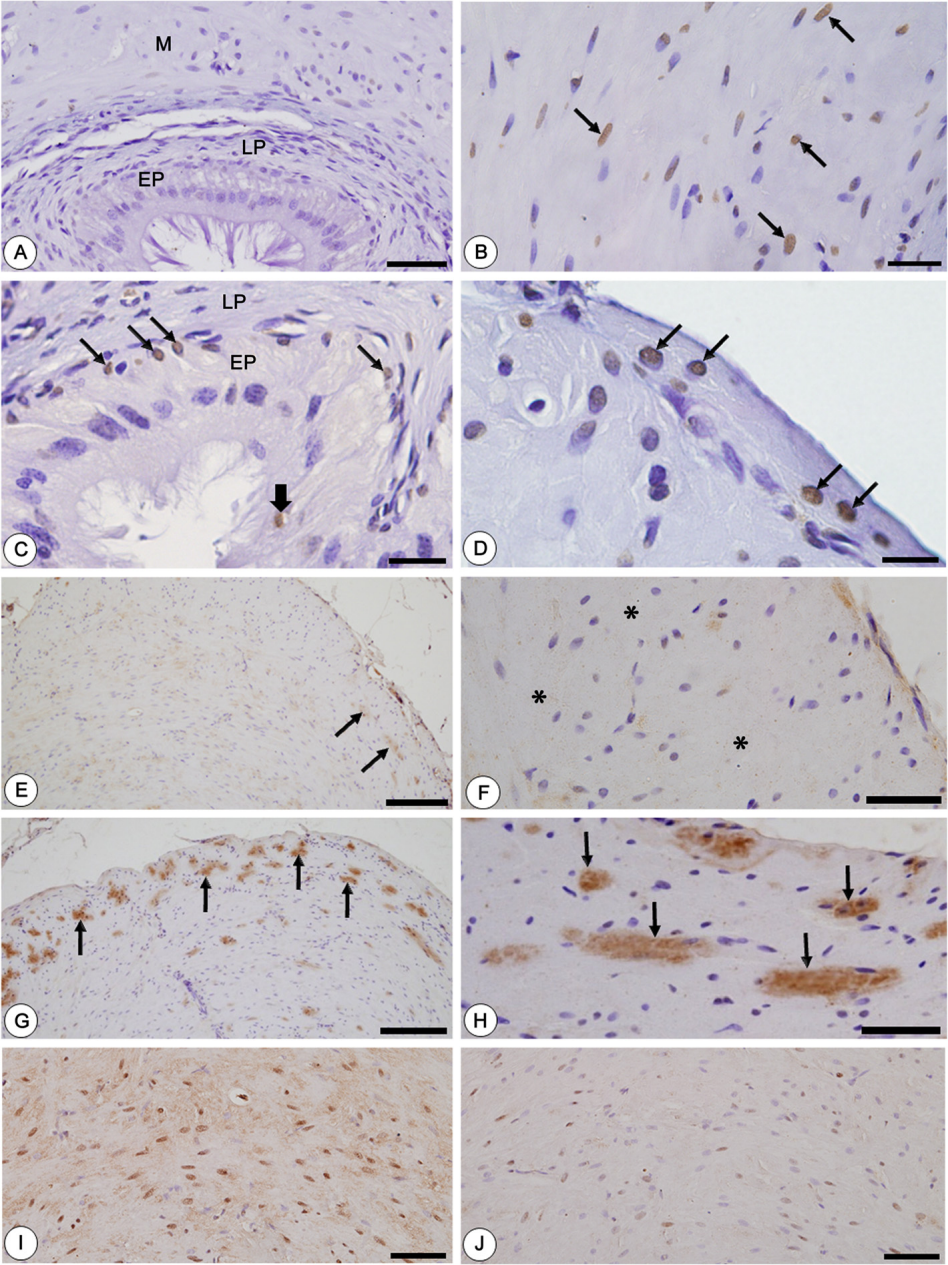
**Photomicrographs of vas deferens of animals from CG (A, E, F, I) and CMTG (B-D, G-H, J) submitted to TUNEL method (A-D), NF-kB (E-H) and AR (I, J) immunohistochemistry and counterstained by hematoxylin.** In **A**, no TUNEL-labeled cells are observed in the vas deferens. However, in **C**, TUNEL-positive nuclei are observed in the columnar (thick arrow) and mainly in the basal (thin arrows) cells of epithelium (EP). Lamina propria (LP). In **B** and **D**, numerous TUNEL-positive muscle cells are also observed in the muscular layer (arrows). In **E** and **F** (CG), portions of the muscular layer show weak (**E**, arrows) or no (**F**; asterisks) NF-kB immunolabeling while in **G** and **H** (CMTG), a strong immunostaining is noted in the cytoplasm of muscle cells (arrows). In **I** and **J**, note the strong (**I**) and weak (**J**) AR immunolabeling in the muscle cells of vas deferens of rats from CG and CMTG, respectively. Bars: 50 μm **(A)**; 20 μm **(B)**; 27 μm **(C)**; 12 μm **(D)**; 156 μm **(E**, **G)**; 38 μm **(F**, **H)**; 58 μm **(I**,**J)**.

In comparison to CG (Figure 2A), numerous TUNEL-positive nuclei were observed in the epithelial cells, mainly in the basal cells (Figure 2C), and also in the muscular layer of vas deferens (Figures 2B and 2D) in all animals from CMTG. The sections of mammary gland (positive control) showed several TUNEL-positive structures. In contrast, no labeling was found in the vas deferens sections used as negative controls (data not illustrated).

### NF-kB and AR immunolabeling

A weak (Figure 2E) or absent (Figure 2F) NF-kB immunostaining was observed in the muscle cells of CG. However, an enhanced NF-kB immunoexpression was detected in the cytoplasm of numerous smooth muscle cells of vas deferens of all rats from CMTG (Figures 2G and 2H). The immunoexpression of AR was inversely proportional to NF-kB; thus, while an enhanced labeling was observed in CG (Figure 2I), a weak immunoexpression was detected in the muscle cells of vas deferens from CMTG (Figure 2J). In the negative controls for NF-kB and AR, no labeling was found (data not illustrated).

### Transmission Electron Microscopy (TEM)

**Figure 3:**
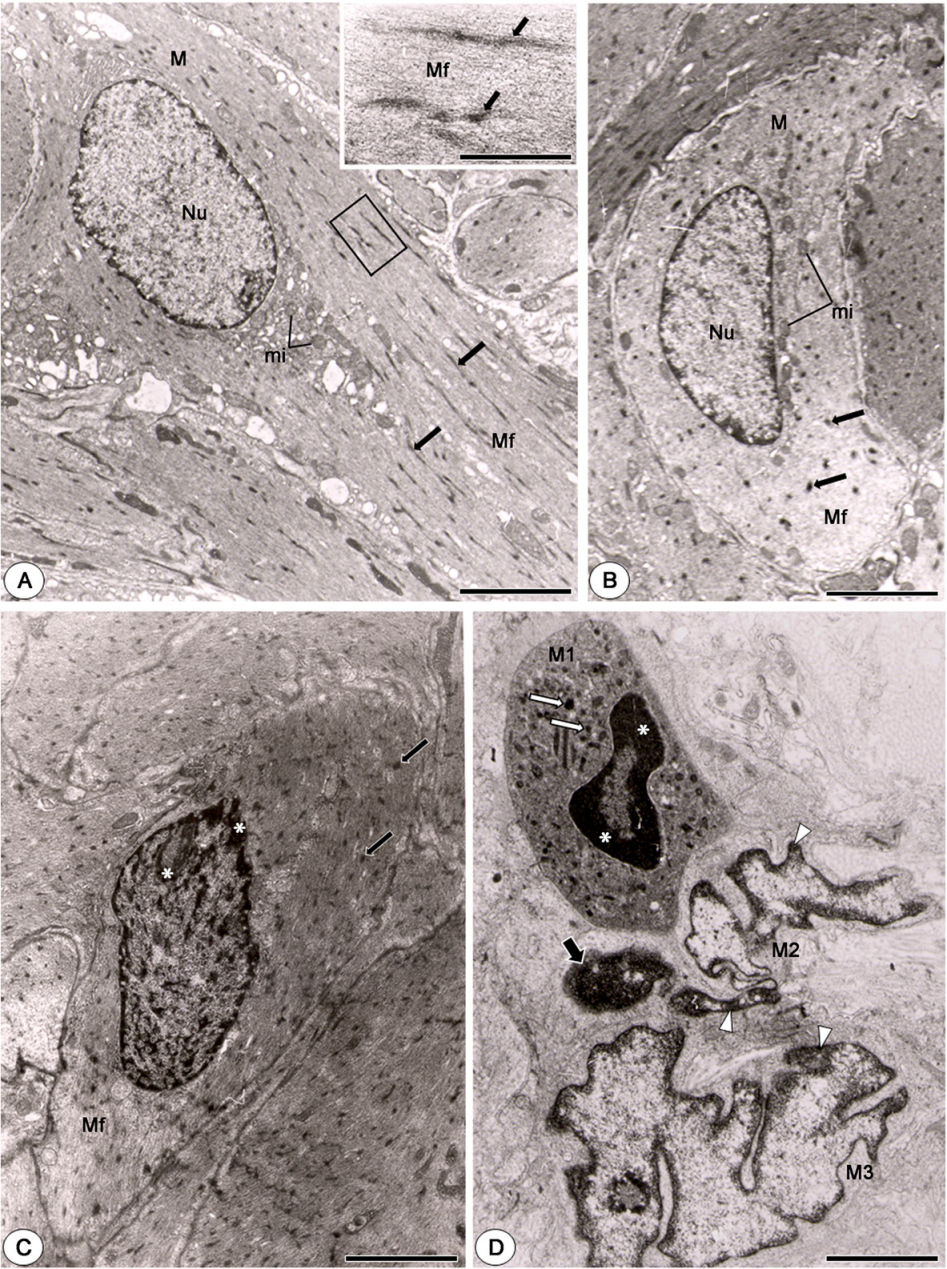
**Electron micrographs of muscular layer of vas deferens of animals from CG(A and B)and CMTG(C and D).** In **A** and **B**, the smooth muscle cells (M) show nucleus (Nu) with regularly distributed chromatin and cytoplasm with numerous mitochondria (mi), miofilaments (Mf) and dense bodies (arrows). In high magnification, miofilaments (Mf) associated to dense bodies (arrows) are observed. In **C**, a cross-sectioned smooth muscle cell with numerous miofilaments (Mf) and dense bodies (arrows) is observed. Note that the cytoplasm is apparently more electron dense than in CG; the nucleus shows portions of strongly condensed chromatin (asterisks). In **D**, the shrunken cytoplasm of a smooth muscle cell (M1) containing dense bodies (white arrows) is more electron dense than CG. The nucleus is irregularly outlined and shows strongly electron dense chromatin in the nuclear periphery (asterisks). Next to this cell, two smooth muscle cells (M2 and M3) with irregular nuclei and portions of chromatin irregularly distributed in the nuclear periphery (arrowheads) are observed. A portion of strongly condensed chromatin is observed close to these nuclei (thick arrow). Bars: 4 μm **(A)**; 1 μm (**A**, inset); 2.5 μm **(B)**; 2 μm **(C** and **D)**.

The smooth muscle cells of vas deferens of animals from CG showed elongate nucleus with regular outline and small clumps of condensed chromatin homogeneously distributed in the nuclear periphery. Mitochondria, myofilaments and dense bodies - typical of smooth muscle cells - were often observed in these cells (Figures 3A and 3B). In CMTG, the muscle cells showed abnormal shape and enhanced cytoplasmic electrondensity due to cellular shrinkage. In these cells, the irregular nuclei showed clumps of electrondense chromatin in the nuclear periphery (Figures 3C and 3D). Portions of strongly electrondense chromatin, probably nuclear fragments, were observed near the nuclei (Figure 3D).

### Morphometrical results

A significant reduction in the epithelial perimeter (21%) and area of the muscular layer (22.5%) was observed in the vas deferens of animals from CMTG in comparison to CG (Table 1).

**Table 1:** **Epithelial perimeter** (**EPer**) **and area of muscular layer** (**MA**) **of vas deferens of rats from CG and CMTG**

**Animals**	**EPer (μm)**	**MA (μm**^**2**^**)**
**CG1**	2,255	2,367
**CG2**	2,427	2,164
**CG3**	1,954	1,969
**CG4**	1,989	2,168
**CG5**	2,077	2,105
**Mean ± SD**	2,140 ± 198	2,154 ± 143
**CMTG1**	1,993	1,705
**CMTG2**	1,721	2,009
**CMTG3**	1,459	1,734
**CMTG4**	1,477	1,594
**CMTG5**	1,800	1,308
**Mean ± SD**	1,690 ± 225*	1,670 ± 253*

*p≤0.05.

### Collagen content under polarized light

**Figure 4:**
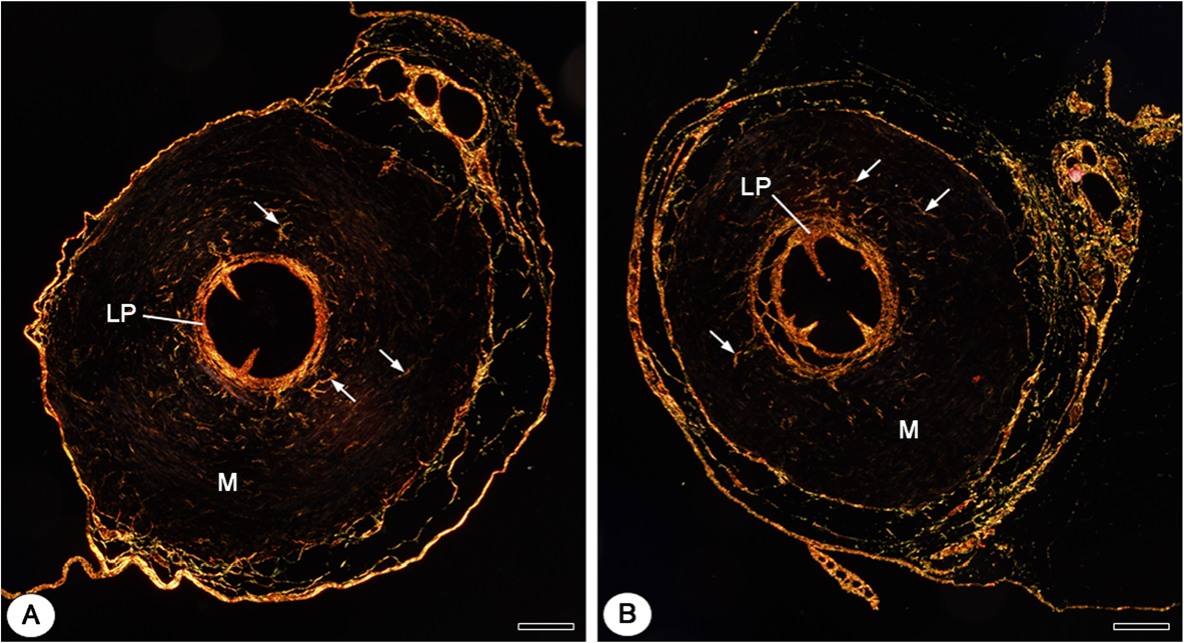
**Photomicrographs of vas deferens of animals from CG (A) and CMTG (B) subjected to Picrosirius-polarization method.** In **A** and **B**, the evident birefringent lamina propria is observed (LP). Note that in both groups **(A** and **B)**, scarce birefringent collagen fibers (arrows) are observed in the muscular layer of vas deferens (M). Bars: 154 μm **(A**, **B)**.

**Figure 5:**
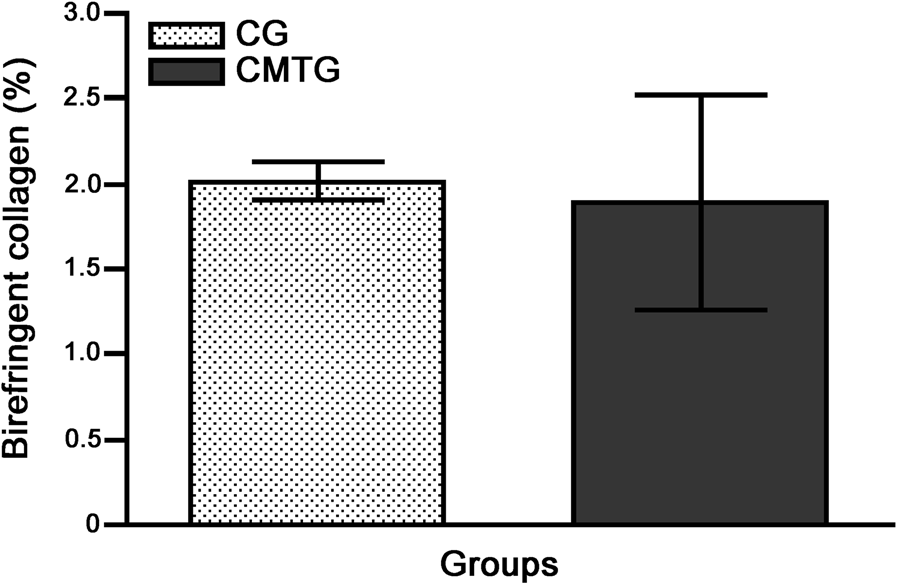
**Percentage of birefringent collagen fibers in the muscular layer of vas deferens of rats from CG and CMTG groups.** No difference is observed between the groups.

The picrosirius-red stained sections of vas deferens under polarized light showed an evident birefringent lamina propria, due to the collagen rich connective tissue, and some sparse birefringent collagen fibers in the muscular layer either in CG or CMTG (Figures 4A and 4B). According to Figure 5, no statistically significant difference in the birefringent collagen fibers percentage was detected in the muscular layer between the groups.

## Discussion

The results showed that cimetidine causes significant reductions in the perimeter of epithelium and area of the smooth muscle layer of vas deferens. The reduction in the epithelial perimeter could be due to death of epithelial cells which was confirmed by the presence of nuclei with condensed chromatin and also labeled by TUNEL, mainly in the basal cells. According to Paniagua *et al.*[37], the basal cells are able to differentiate into the different columnar cells, playing a role in the epithelial turnover. Therefore, the significant reduction in the epithelial perimeter may be due, at least in part, to a possible interference of cimetidine on the basal epithelial cells, leading to a total decrease in the cellular population. The mechanism by which cimetidine induces epithelial cell death in the vas deferens should be further investigated. It is known that cimetidine inhibits ARs in different tissues that require androgens [17-19]. However, studies have demonstrated that androgens are essential for the structural and functional maintenance of the vas deferens. In castrate mice, Hamilton *et al.*[2] demonstrated alterations in the epithelial cells of vas deferens such as absence of smooth endoplasmic reticulum, reduction in the size of the Golgi apparatus and decreased number of mitochondria. In mammals, including rodents [1], ARs have been detected in the epithelial cells of vas deferens [38,39]. According to Zhou *et al.*[1], the basal cells show a more accentuated AR immunoexpression in comparison to the other epithelial cells. Moreover, alterations in the basal cells of rat vas deferens caused by suppression of testosterone levels confirm that these cells are dependent of androgen [40]. Therefore, the morphological alterations observed in the epithelial layer of CMTG animals such as: reduction of the epithelial perimeter, presence of pyknotic nuclei, TUNEL-positive nuclei and intraepithelial vacuoles may be related to a possible antiandrogenic effect of cimetidine on the epithelial cells.

The smooth muscular layer was also affected by cimetidine treatment since muscle cell death, confirmed by TUNEL and TEM, and a significant reduction of muscular area was detected in CMTG. The reduction in the muscular layer is related to the muscle cells death by apoptosis. Additionally to the TUNEL method, the presence of ultrastructural features typical of apoptosis such as the presence of peripheral condensed chromatin in the nuclei, nuclear fragments and cellular shrinkage [41], indicates that these cells undergo apoptosis. In previous studies, cimetidine has been demonstrated to exert a harmful effect on peritubular myoid cells, [21,24] and also on the smooth muscle cells of testicular blood vessels [25], leading to apoptosis in these cells. Thus, in the present study, the presence of muscle cell death and muscular layer atrophy in the vas deferens confirm the susceptibility of smooth muscle cells to cimetidine treatment. However, the cellular way by which cimetidine induces smooth muscle cell death needs to be clarified. It is possible that the atrophy of vas deferens muscular layer is related to the cimetidine antagonist effect on histamine H_2_ receptors since histamine exerts an inhibitory effect, via H_2_ receptors, on the vas deferens contraction [6,7]. The activation of H_2_ receptors by histamine inhibits the muscle contraction response due to a decrease in the Ca^2+^ influx necessary for contraction. However, this effect (muscle relaxing) is reduced or totally antagonized by cimetidine [8,42], leading to increased intracellular calcium influx. The excess of calcium ions seems to be one of the main routes involved in the induction of programmed cell death [43,44]. It has been demonstrated that simvastatin induces apoptosis in the smooth muscle cells due to increased Ca^2+^ levels and, subsequently, caspase activation [45]. Therefore, additionally to a possible antiandrogenic effect of cimetidine (as discussed below), it is possible that smooth muscle cell death of vas deferens is caused by increased intracellular calcium influx due to cimetidine antagonist action on H_2_ receptors. Although cimetidine also exerts antiandrogenic and antiangiogenic actions, further studies focusing on the comparison of cimetidine effects with other histamine H_2_ receptors antagonist effects would be advisable.

Besides H_2_ receptors, ARs have also been detected in the smooth muscle cells of vas deferens [1] and testicular arterioles [46]. Type L calcium channels of smooth muscle cells are inhibited by testosterone [3,4]. In the rat vas deferens, 5α-DHT inhibits Ca^2+^ influx through the voltage-dependent calcium channels and, then, inhibits smooth muscle contraction [5]. As caspase-stimulated smooth muscle cell apoptosis occurs by increased calcium influx [43,44], the muscular layer atrophy of vas deferens following cimetidine treatment could also be related to the antiandrogenic effect of this drug on the smooth muscle cells.

Another point to be emphasized is that the high incidence of TUNEL-positive cells was parallel to the overexpression of NF-kB in the muscular layer of rat vas deferens from CMTG. NF-kB transcription and activation play a crucial role in regulating the process of cell death by apoptosis in several cell types including smooth muscle cells [25,31]. In most cell types, NF-kB remains bound to IkBα protein and thereby is inactive in the cytoplasm [47,48]. After stimulation by various reagents, IkBα is rapidly phosphorylated by the IkB kinase (IKK) complex and degraded by the proteasome, allowing NF-kB to translocate to the nucleus and activate its target gene [27,49,50]. Thus, NF-kB activates the transcription of many genes capable of suppressing cell death [30]. Studies have demonstrated that vascular smooth muscle cells apoptosis has been commonly observed in response to inhibition of NF-kB by IkBα [31], by Propionyl L-carnitine [51] and also cimetidine. This drug (cimetidine) inhibits the translocation of NF-kB to the nucleus, decreasing the transcription of antiapoptotic genes and inducing apoptosis in salivary gland tumor cells [32]. Pretreatment with roxatidine (anti-H_2_ receptor and AR antagonist) has also demonstrated to inhibit the translocation of the activated NF-kB subunits, p65 and p50, to the nucleus [52]. These findings are consonant to our results since an enhanced NF-kB immunoexpression was found in the cytoplasm, but not nuclei, of smooth muscle cells following cimetidine treatment. A similar increased immunolabeling has also been demonstrated in a study, in which NF-kB p50 was used, and has been related to a possible epitope unmasking [53]. The antibody used in the present study bounds around to phosphorylation site of Serine 276 that belongs to the amino acids sequence 1–313 of p65. This sequence containing the nuclear localization signal is essential for IkBα binding [54]. Therefore, the enhanced NF-kB immunostaining in the muscle cells of cimetidine treated rats could be resulted from the unmasking epitope and indicates that this factor is in its active form in the cytoplasm.

The decreased AR immunolabeling in the cimetidine-treated rats also reinforces the idea that cimetidine may have interfered in the translocation of the activated NF-kB to the nucleus. AR gene is a NF-kB target gene; thus, overexpression of NF-kB factor p65 causes increased AR mRNA and protein levels [55]. Therefore, our results indicate that muscle cell apoptosis can be related to a possible interference of cimetidine on the translocation of the activated NF-kB to the nucleus. A plausible explanation for this interference can be related to the antiandrogenic action (AR antagonist) of cimetidine since studies focusing on the effects of AR on NF-kB activity in the prostate cancer cell lines have demonstrated that NF-kB activity depends on the availability of androgens. Thus, in the presence of androgens, NF-kB activity is decreased by AR while in the absence of ligand, AR increases NF-kB activity [56].

Regarding the collagen content, smooth muscle tissue substitution by collagen fibers (fibrous tissue) has been detected in the smooth muscular wall of vas deferens of dogs and rats subjected to different injuries [57-59]. In the present study, it was supposed that an increase of fibrous tissue (collagen fibers) would occur among the muscle cells during smooth muscular layer involution. However, no collagen content difference was detected between the groups after the quantitative analysis of the birefringent collagen under polarized light. This finding can explain the significant reduction of the muscular area. The absence of fibrosis during the involution of muscular layer, observed in the present study, could be related to a possible histamine antagonist effect of cimetidine on the fibroblasts. This is reinforced by the fact that histamine stimulates fibroblasts proliferation via H_2_ receptors [60]. Moreover, type I collagen synthesis by fibroblasts, stimulated by histamine via H_2_ receptors, is inhibited by cimetidine [61]. However, further studies are necessary to confirm this possibility.

## Conclusions

In conclusion, the epithelial alterations caused by cimetidine in the vas deferens seem to be related to an interference of this drug on the epithelial turnover due to a possible antiandrogenic effect. The significant reduction in the muscular layer caused by muscle cells apoptosis confirms the susceptibility of smooth muscle cells to the cimetidine treatment. A possible interference of the drug on the histamine and/or androgen-mediated calcium influx should be further investigated. The enhanced immunolabeling of NF-kB in the cytoplasm of smooth muscle cells following cimetidine treatment indicates that muscle cell apoptosis may be caused by an interference of cimetidine on the translocation of active NF-kB to the nucleus. Considering that NF-kB induces AR expression, the decreased immunoexpression of ARs verified in the muscle cells reinforces this interference. It is possible that these effects are derived from antiandrogenic action of cimetidine. However, further studies focusing on the comparison of these results with effects of other antiandrogenic drugs and H_2_ receptor antagonists are necessary to confirm this possibility.

## Competing interests

The authors declare that they have no competing interests.

## Authors’ contributions

ESC coordinated the study. JYK, FLB and BHC carried out the treatments of animals. JYK performed the histological processing, morphometric and statistical analyzes. JYK, BHC and PSC prepared the specimens for analysis under TEM and PSC obtained the TEM images. JYK, FLB, BHC and ESC performed the TUNEL and immunohistochemical reactions. JPPJr. carried out the collagen quantitative analysis under polarized light. All authors selected the images and participated in the design and writing of the manuscript. All authors read and approved the final manuscript.
